# Usefulness of monitoring circulating tumor cells as a therapeutic biomarker in melanoma with *BRAF* mutation

**DOI:** 10.1186/s12885-021-08016-y

**Published:** 2021-03-17

**Authors:** Yukiko Kiniwa, Kenta Nakamura, Asuka Mikoshiba, Atsuko Ashida, Yasuyuki Akiyama, Atsushi Morimoto, Ryuhei Okuyama

**Affiliations:** 1 Department of Dermatology, Shinshu University School of Medicine, 3-1-1 Asahi, Matsumoto, Nagano 390-8621 Japan; 2 Life Science Research Laboratory, Tosoh Corporation, Ayase, Kanagawa, Japan

**Keywords:** *BRAF*, Circulating tumor cell, Melanoma, BRAF/MEK inhibitor, Heterogenous mutation

## Abstract

**Background:**

While molecularly targeted therapies and immune checkpoint inhibitors have improved the prognosis of advanced melanoma, biomarkers are required to monitor drug responses. Circulating tumor cells (CTCs) are released from primary and/or metastatic tumors into the peripheral blood. We examined whether CTCs have potential as biomarkers by checking the number of CTCs, as well as the *BRAF* genotype of individual CTCs, in melanoma patients undergoing BRAF/MEK inhibitor treatment.

**Methods:**

CTCs were isolated from peripheral blood using a high-density dielectrophoretic microwell array, followed by labeling with melanoma-specific markers (MART-1 and/or gp100) and a leukocyte marker (CD45). The numbers of CTCs were analyzed in fifteen patients with stage 0–III melanoma. Furthermore, changes in CTC numbers were assessed in five patients with stage IV melanoma at four time points during BRAF/MEK inhibitor treatment, and the *BRAF* genotype was analyzed in CTCs isolated from one patient.

**Results:**

We examined CTCs in patients with stage 0–III (five samples per stage: stage 0–I, stage II, and stage III), and detected CTCs even in patients with early disease (stage 0 and I). Interestingly, recurrence occurred in the lymph nodes of one stage I patient 2 years after the detection of a high number of CTCs in the patient’s blood. The total number of CTCs in four of five patients with stage IV melanoma fluctuated in response to BRAF/MEK inhibitor treatment, suggesting that CTC number has potential for use as a drug response marker in advanced disease patients. Interestingly, one of those patients had CTCs harboring seven different *BRAF* genotypes, and the mutated CTCs disappeared upon BRAF/MEK inhibitor treatment, except for those harboring *BRAF*^A598V^.

**Conclusions:**

CTCs are present even in the early stage of melanoma, and the number of CTCs seems to reflect patients’ responses to BRAF/MEK inhibitor treatment. Furthermore, genetic heterogeneity of *BRAF* may contribute to resistance to BRAF/MEK inhibitors. Our findings demonstrate the usefulness of CTC analysis for monitoring responses to targeted therapies in melanoma patients, and for understanding the mechanism of drug resistance.

**Supplementary Information:**

The online version contains supplementary material available at 10.1186/s12885-021-08016-y.

## Background

Molecularly targeted therapies and immune checkpoint inhibitors have improved the prognosis of advanced melanoma. Although the objective response rates to those treatments range from 40 to 70% in clinical trials [1], real-world outcomes are inferior [2]. Therefore, prediction of drug response and optimization of treatment order are required. Pretreatment tumor biopsies provide useful information, including driver mutations, expression levels of programmed death-ligand 1, infiltration of CD8-positive T-cells within tumors, and microsatellite instability. However, those markers are insufficient for predicting response to treatment because they reflect tumor status at a single time point. Moreover, although additional biopsies may be desired during treatment, tissue biopsies may not accurately reflect systemic tumor status due to intertumoral and intratumoral heterogeneity [3].

Baseline factors, including lactate dehydrogenase (LDH) levels, the number of metastatic organs, and Eastern Cooperative Oncology Group performance status, have been reported to be well-correlated with survival in clinical trials for advanced melanoma [4]. However, LDH levels also reflect the side effects of drugs and infection; thus, they may not suffice as an accurate biomarker of disease status. Liquid biopsy, including circulating tumor cells (CTCs), circulating tumor DNA (ctDNA), and circulating microRNA, have attracted attention as potential biomarkers [5–7]. ctDNA, which is released from dead tumor cells, is present in peripheral blood as cell-free DNA and is a useful tool for monitoring real-time disease status. Although ctDNA is quite specific for tumors, it is rapidly degraded, decreasing the sensitivity of assays that are based on it. In addition, some mutated cell-free DNA is produced in clonal hematopoiesis [7].

CTCs are released from primary and/or metastatic tumors into the peripheral blood. Interestingly, an increase in the number of pulmonary venous CTCs at the time of surgery for early-stage non-small-cell lung cancer is associated with disease relapse, suggesting that early-disseminating tumor cells in regional veins are responsible for the relapse [8]. Several strategies have been used to detect CTC, including a microbead-sorting method, flow cytometry, microfluidics, and filtration-based devices [9–12]. In combination with negative selection for leukocyte specific markers, various markers are used to detect melanoma cells in peripheral blood, including CD146, melanoma-associated chondroitin sulfate proteoglycan, ATP-binding cassette subfamily B member 5, CD271, and receptor activator of NF-κB. Assuming that the CTC population represents the distributed tumor burden and biological features, characterization of these cells could provide a complementary sample for the monitoring of tumor characteristics [12–14]. CTCs are a promising source of material because they can be obtained via routine blood sampling and can provide real-time information about the characteristics of tumors over time. CTC characterization can reveal the early response to immune checkpoint inhibitors in melanoma [15] and identify genetic heterogeneity in *BRAF* V600 status [16]. Furthermore, an increase in the number of pre-operative CTCs in melanoma patients with regional lymph node (LN) metastasis is associated with the risk of recurrence after LN dissection [17], suggesting adjuvant therapies may be needed in patients with high numbers of CTCs before dissection.

According to recent long-term observations, patients treated with combinations of BRAF/MEK inhibitors exhibit favorable outcomes. In particular, patients with complete remission achieve longer progression-free survival and overall survival [4]. However, the majority of patients with partial response or stable disease exhibit a short-duration response and experience recurrence within several months after initiation of therapy. Therefore, it is necessary to establish biomarkers that enable early detection of recurrence and evaluation of treatment response. To this end, as well as to elucidate the mechanisms of drug resistance, analysis of CTCs may be useful. Hence, in this study, we monitored the number of CTCs along with the *BRAF* genotype during treatment with BRAF/MEK inhibitors.

## Methods

### Blood and tissue samples

Peripheral blood was obtained from patients with melanoma and from healthy individuals. For CTC analysis of stage 0–III melanoma patients, five samples per stage (stage 0–I, stage II, and stage III) were collected before surgical resection of the primary tumor and sentinel node biopsy. For CTC analysis of metastatic melanoma patients, blood was collected once before treatment and at any three time points during BRAF targeted therapy. CTC samples were collected randomly during otherwise routine clinic visits. Formalin-fixed paraffin-embedded tissues were used for pathological diagnosis and *BRAF* V600 genotyping. When applied to the primary tumor biopsies, the Cobas 4800 BRAF Mutation Test (Roche Molecular Diagnosis, Basel, Switzerland) or the Oncomine Dx Target Test (Thermo Scientific, Waltham, MA, USA) was positive in all metastatic patients treated with BRAF/MEK inhibitors (Table S1).

### Identification of CTC

To analyze tumor features, we monitored CTCs using a high-density dielectrophoretic microwell array. The principles underlying identification and capture of CTCs were described previously [18]. In brief, peripheral blood mononuclear cells were resuspended in 300 mM mannitol solution, a solution with suitable conductivity for dielectrophoresis. The suspension was loaded into the cell entrapment chamber, and the cells were entrapped in the microwells by dielectrophoretic force. The trapped cells were labeled with antibodies against the melanoma-specific markers MART-1 (BioLegend, San Diego, CA, USA) and gp100 (DAKO, Santa Clara, CA, USA), followed by anti-mouse IgG antibody conjugated to Alexa Fluor 488 (Life Technologies, Eugene, OR, USA). To exclude leukocytes, we used an anti-CD45 antibody conjugated to phycoerythrin (Beckman Coulter, Marseille, France). Subsequently, fluorescence microscopy was used to capture images of the cells entrapped in each well. MART-1/gp100-positive and CD45-negative cells were counted as CTCs. In addition, a spike-in experiment was performed, the results of which are shown in Supporting Information. Finally, in *BRAF*^V600E/K^ patients, CTCs were captured by micromanipulation and subjected to DNA sequencing.

### Isolation and mutation analysis of CTC

For single-cell sequencing of CTCs, captured cells from the cell entrapment chambers were singly collected in tubes containing 20 μL nuclease-free water. For DNA sequencing, genomic DNA was extracted from each cell, followed by PCR amplification of *BRAF* exon 15 and Sanger sequencing as described previously [19]. Detailed information on DNA sequencing is presented in Supporting Information.

### Statistical analysis

For statistical analysis, the Student’s *t*-test was used to compare the number of CTCs between patients with different stages. Differences and correlations were considered significant when *p* < 0.05.

## Results

### CTC detection in patients with stage 0–III melanoma

**Fig. 1:**
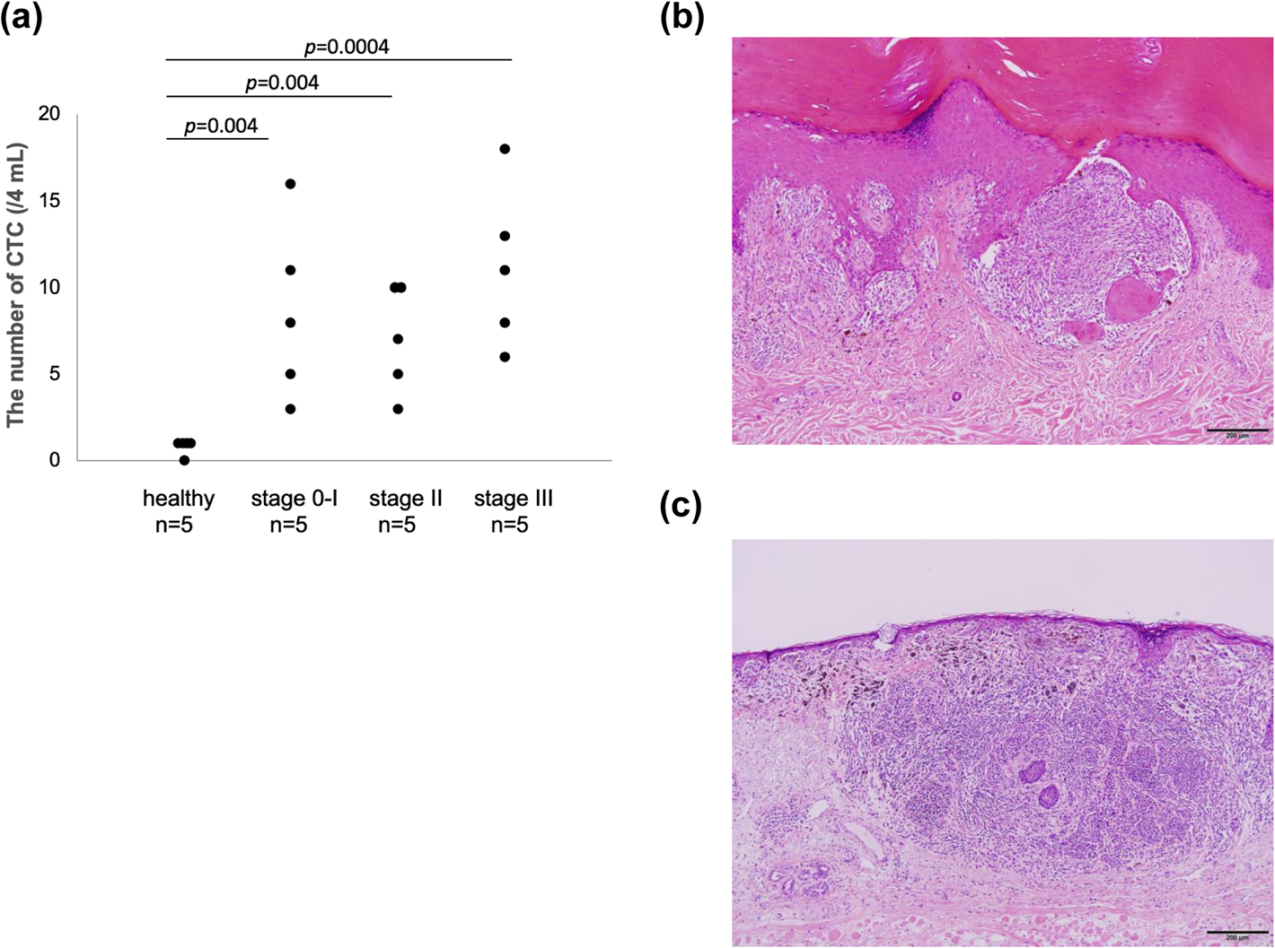
Number of circulating tumor cells (CTCs) at stages 0–I, II, and III. **a** Number of CTCs in healthy individuals and melanoma patients at stages 0–I, II, and III. CTCs were counted using blood samples collected before surgical resection of the primary tumor and sentinel node biopsy. **b**, **c** Pathologic features of stage I primary melanoma. Atypical melanocytes invaded the dermis in a nodular and diffuse manner. Tumor thicknesses were 0.6 (**b**) and 0.8 mm (**c**). Clark levels were III (**b**) and IV (**c**) (× 100, hematoxylin/eosin staining)

Peripheral blood was collected from melanoma patients with stage 0–III disease before surgical resection of the primary tumor and sentinel node biopsy (Table S2). Melanoma stage was determined based on the American Joint Committee on Cancer Staging Manual (8th edition). To distinguish tumor cells from white blood cells, CTCs were defined as positive for melanoma-specific markers (MART-1 and/or gp100) and negative for CD45. We defined the intensity threshold of each parameter to minimize false positivity, using a mixture of melanoma cell lines with normal blood cells. Sensitivity and specificity were 12.6–60.6% (Fig. S1) and 99.9% (data not shown), respectively; sensitivity differed among cell lines. For CTC analysis of stage 0–III melanoma patients, five samples per stage (stage 0–I, stage II, and stage III) were analyzed. Five samples from healthy individuals were also collected and analyzed. The number of CTCs per 4 mL blood in stage 0–I, stage II, and stage III disease was 3–16 (median, 8; interquartile range, 6), 3–10 (median, 7; interquartile range, 5), and 6–18 (median, 11; interquartile range, 5), respectively (Fig. 1a; Table S2). The number of CTCs was not well correlated with tumor thickness. It is unclear whether the number is related to recurrence or clinical prognosis. Interestingly, bulky recurrence occurred 2 years after blood collection in the lymph nodes of a stage I patient who had a high number of CTCs (16 CTCs per 4 mL blood). In addition, we detected five cells per 4 mL blood in a patient with melanoma in situ. By contrast, zero or one cell meeting the criteria for CTCs was present per 4 mL blood in healthy individuals. In primary tumors (stage I), bulky nests of melanoma cells in the dermis may have been the source of CTCs (Fig. 1b, c).

### Monitoring CTCs in *BRAF*-mutated advanced melanoma

To evaluate the usefulness of CTCs as biomarkers of responsiveness to treatment, we next analyzed blood from five patients with *BRAF*-mutated melanoma (MMbraf1–5), who were treated with BRAF/MEK inhibitors (Table S1). Objective response to therapies was assessed by computed tomography (CT) scan using the Response Evaluation Criteria in Solid Tumors (RECIST) version 1.1 (CR, complete response; PR, partial response; SD, stable disease; and PD, progressive disease).

**Fig. 2:**
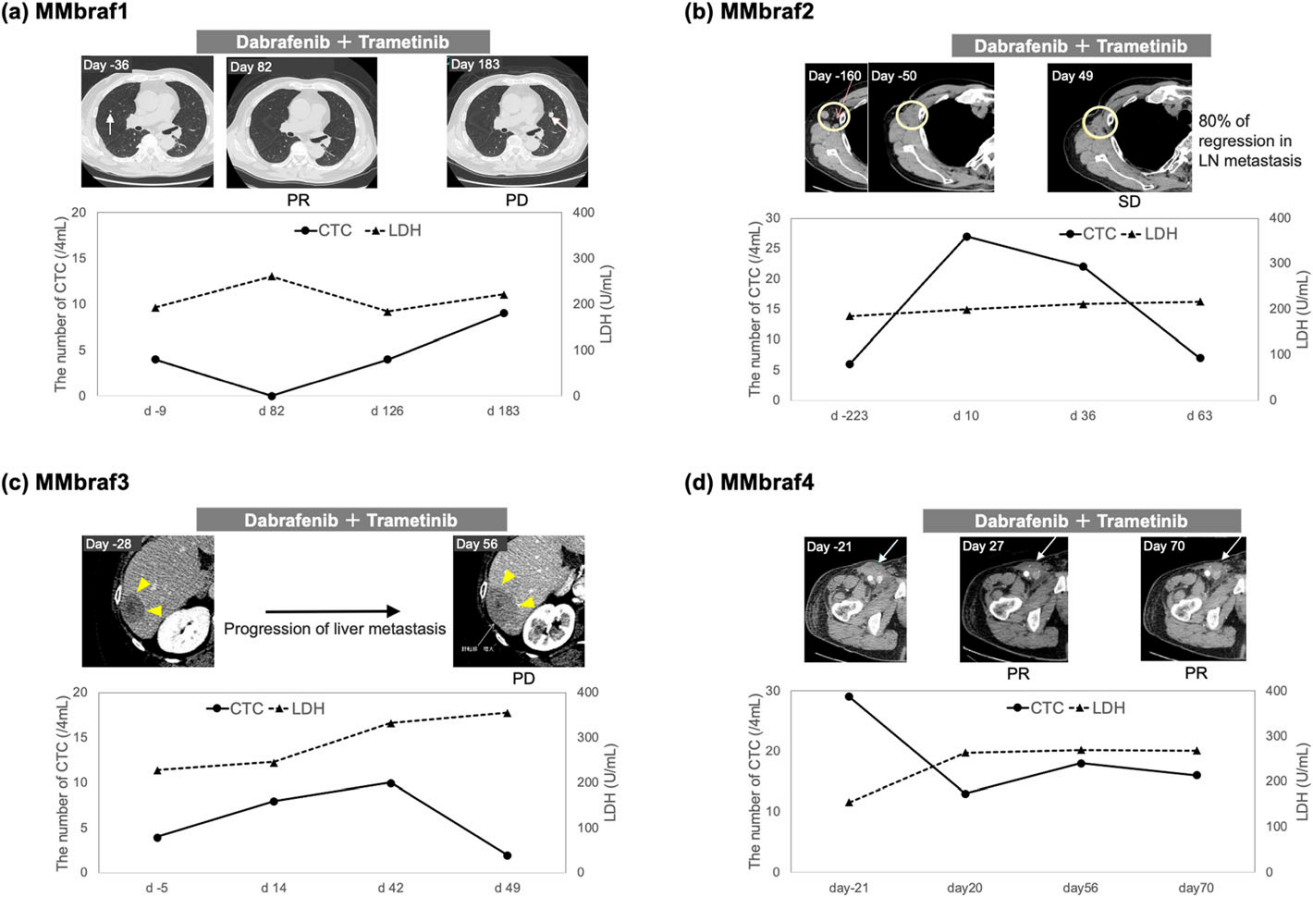
Number of CTCs during treatment with BRAF/MEK inhibitors. MMbraf1, MMbraf2, MMbraf3, and MMbraf4 were diagnosed with metastatic *BRAF*^v600E/K^ melanoma. **a** Monitoring the number of CTCs during the clinical course in MMbraf1. The graph shows the number of CTCs and the LDH level (upper limit of normal, 230 IU/L). Arrows indicate lung metastases in computed tomography (CT) imaging. **b** Monitoring the number of CTCs during the clinical course in MMbraf2. Circles indicate a right axillary lymph node (LN) metastasis in CT imaging. **c** Monitoring the number of CTCs during the clinical course in MMbraf3. Arrowheads indicate a liver metastasis in CT imaging. **d** Monitoring the number of CTCs during the clinical course in MMbraf4. Arrows indicate a right inguinal LN metastasis in CT imaging

MMbraf1–4 were treated with dabrafenib and trametinib for unresectable metastases. We monitored the number of CTCs at four time points. CTC samplings were collected randomly during otherwise routine clinic visits. In MMbraf1, administration of dabrafenib and trametinib (Day 0) resulted in a decrease in the number of CTCs on Day 82, and lactate dehydrogenase (LDH; upper limit of normal, 230 U/L) levels increased moderately at the same timepoint (Fig. 2a). A CT scan revealed a significant reduction in tumor size, and tumor response was classified as PR. Subsequently, the number of CTCs increased on Day 126, and on Day 183 the tumor response was categorized as PD based on the appearance of a novel metastatic lesion in the left lung. In MMbraf2, the number of CTCs began to decrease on Day 36, and the metastatic lesion had partially regressed on Day 49, corresponding to SD (Fig. 2b). LDH levels increased slightly at the same timepoint. In MMbraf3, the number of CTCs increased on Days 14 and 42, but suddenly decreased on Day 49 (Fig. 2c). LDH levels increased at these timepoints, and CT scan revealed enlargement of a metastatic lesion in the liver on Day 56, corresponding to PD. In MMbraf4, CTCs were less abundant on Days 20, 56, and 70 than at the beginning (Fig. 2d). The LDH level increased slightly on Day 20. Thereafter, although LDH level was stable, it remained above the upper limit of the normal range. A CT scan revealed PR on Day 70.

### Monitoring *BRAF*-mutated CTCs during BRAF targeted therapy

**Fig. 3:**
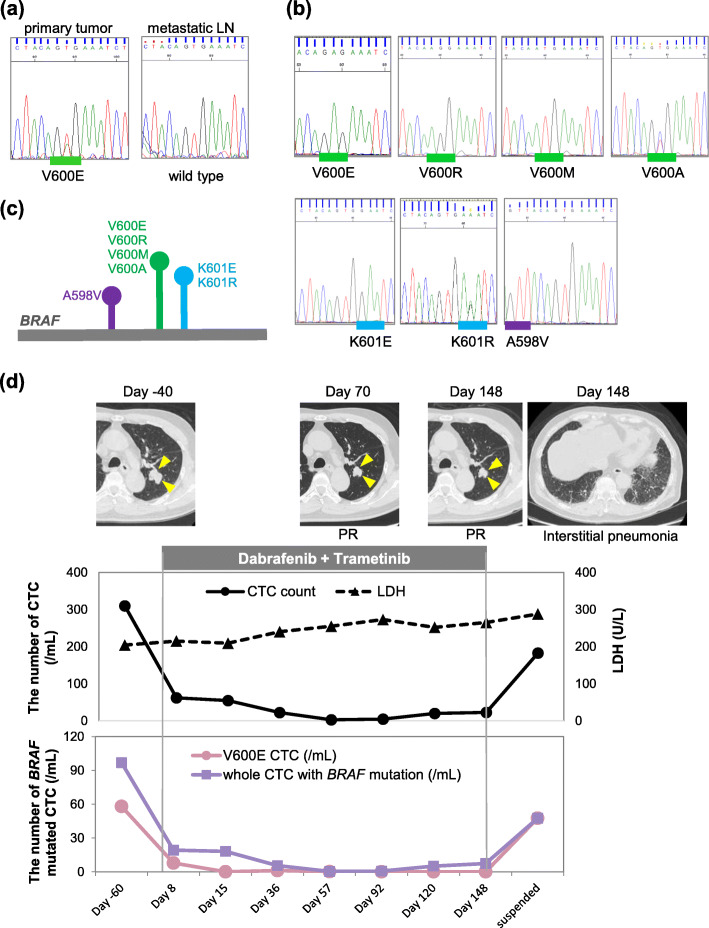
*BRAF* genotype and the number of CTCs in MMbraf5. **a** *BRAF* sequence chromatograms of the primary tumor and a metastatic lymph node. **b** *BRAF* sequence chromatograms of CTCs during treatment with BRAF/MEK inhibitors. **c** Diversity of *BRAF* mutations surrounding codon 600 in MMbraf5. **d** Number of CTCs during treatment and clinical outcome response in patient MMbraf5. Arrowheads indicate lung metastasis in CT imaging on Days − 40, 70, and 148. Far-right CT imaging shows the appearance of interstitial pneumonia on Day 148. In the upper graph, solid and dotted lines indicate the number of total CTCs and the LDH level, respectively, during the clinical course in MMbraf5. In the lower graph, pink and purple lines indicate the numbers of *BRAF*^V600E^ CTCs and total CTCs with *BRAF* mutations, respectively

In MMbraf5, *BRAF*^V600E^ mutation was identified in a primary tumor but not in a LN metastasis (Fig. 3a), suggesting heterogeneity of the *BRAF* genotype. Therefore, we decided to investigate the *BRAF* genotype of CTCs at the single-cell level. When a lung metastasis was detected by CT scan, the patient was initially treated with nivolumab. Because the lung metastasis was enlarged 9 months later, nivolumab was switched to dabrafenib and trametinib. Before switching the therapy, the total number of CTCs was 310 /mL with 58.1 /mL *BRAF*^V600E^-mutated CTCs. On Day 8, the numbers of both total and *BRAF*^V600E^-mutated CTCs decreased to 62 /mL and 5 /mL, respectively (Fig. 3b, c, d; Table 1). In addition, *BRAF*^V600R^, *BRAF*^V600M^, *BRAF*^V600A^, *BRAF*^K601E^, *BRAF*^K601R^, and *BRAF*^A598V^ CTCs were found in the blood (Fig. 3b, c). After initiation of dabrafenib and trametinib, *BRAF*^V600E^ CTCs gradually decreased and finally disappeared on Day 92 (Fig. 3d). Similarly, *BRAF*^V600R^, *BRAF*^V600M^, *BRAF*^V600A^, *BRAF*^K601E^, and *BRAF*^K601R^ CTCs disappeared until Day 120. On the other hand, the number of total CTCs decreased but never disappeared. Interestingly, *BRAF*^A598V^ and *BRAF* wild-type CTCs were still detected even after other *BRAF*-mutated CTCs disappeared (Table 1). A CT scan on Day 70 revealed that tumor response was classified as PR due to a reduction of lung metastasis. By contrast, LDH levels did not decrease during treatment, probably due to an adverse event. Because the patient was diagnosed with drug-induced interstitial pneumonia on Day 148, dabrafenib and trametinib were suspended. Thereafter, *BRAF*^V600E^-mutated CTCs reappeared and the number of total CTCs increased (Fig. 3d; Table 1).

**Table 1:** *BRAF* status of CTCs detected in MMbraf5 during treatment with dabrafenib and trametinib

***BRAF*** mutation status of CTC	Day − 60	Day 8	Day 15	Day 36	Day 57	Day 92	Day 120	Day 148	Treatment suspended
**Wild type**	34	9	14	15	14	6	3	8	17
**V600E**	9	2	2	1	1	0	0	0	6
**V600R**	0	1	5	2	1	0	0	0	0
**V600M**	0	0	0	1	0	0	0	0	0
**V600A**	0	1	0	0	0	0	0	0	0
**K601E**	4	2	0	1	1	1	0	0	0
**K601R**	1	0	0	0	0	0	0	0	0
**A598V**	0	2	0	0	1	0	1	3	0
**The number of isolated CTC**	48	17	21	20	18	7	4	11	23

Day, day before or after treatment started

We did not analyze *BRAF* genotyp of all isolated CTC at each time point

## Discussion

CTCs have been actively studied in the context of solid tumors. Here, we analyzed CTCs in melanoma using a high-density dielectrophoretic microwell array, followed by labeling of CTC with markers specific for melanoma and leukocytes [16, 18]. Because the relatively straightforward assay, from labeling to detection, can be performed on the same plate, this method is useful for isolation and characterization of small numbers of cells at the single-cell level.

Our results demonstrated that CTCs were present even in stage 0 or I melanoma. Although CTCs are present in limited numbers [10], they exist even in the early stages of melanoma, as well as in other diseases such as breast and lung cancer [20–22]. Primary tumors with minimal invasion may exert their metastatic potential via releasing CTCs.

In addition, we found that the number of CTCs was not well correlated with tumor thickness. Tumor cells usually transform to an invasive and metastatic phenotype in response to hypoxia, genetic instability, and activation of oncogenes [23]. Because hypoxia in tumor tissues elicits angiogenesis, the formation of a bulky mass can cause local hypoxia and activate the potential to migrate to and access blood vessels even in early-stage disease. Notably in this regard, we detected CTCs even in a case with melanoma in situ. Theoretically, a melanoma in situ should not metastasize, but when it does, it is likely to be due to occult invasive lesions within the tumor [24, 25]. The presence of such lesions can be revealed by performing serial sectioning deeper into the tumor tissue block.

Although we analyzed a small number of cases, we found that the number of CTCs was not associated with clinical stage, consistent with previous studies of melanoma and lung cancer [12, 20]. Interestingly, detection of CTCs is associated with overall survival in stage II–III patients with melanoma [17]. Thus, the number of CTCs may be a prognostic biomarker among patients with the same staging.

The results of this study demonstrated that alteration of CTC number is associated with a clinical response to BRAF/MEK inhibitors. LDH levels were correlated with clinical response in two out of five patients, and the number of CTCs seemed to reflect the response in four out of five patients, suggesting that CTC count could be a useful biomarker for advanced melanoma treated with BRAF/MEK inhibitors. In a recent study that combined analysis of CTCs and ctDNA, CTC number was strongly associated with the level of ctDNA; moreover, the number of CTCs prior to systemic therapies was negatively correlated with overall and progression-free survival [13]. These observations, along with our results, indicate that detection of CTCs during treatment provides useful information that supports imaging studies, such as CT scans, in the prediction of drug response and prognosis.

Heterogeneity of protein expression and genetic alteration in CTCs has been reported in melanoma [11, 12, 16, 26]. In some patients, CTCs are heterogenous with respect to the *BRAF* genotype [12, 16]. In this study, BRAF/MEK inhibitors seemed to be effective against melanoma cells with various mutations in V600 and K601 residues of BRAF. However, CTCs with *BRAF*^A598V^ persisted throughout treatment, implying a potential mechanism of drug resistance. Future studies should test this possibility.

## Conclusions

CTC analysis is useful for evaluating disease status during molecularly targeted therapies, and analysis at the single-cell level may provide information for overcoming drug resistance. In addition, CTCs with certain properties may develop into metastases, suggesting that analysis of CTCs could shed light on the metastatic signature.

## Supplementary Information

**Additional file 1.**

**Additional file 2.**

**Additional file 3.**

## Data Availability

The datasets used and analyzed during the current study are available from the first author Dr. Yukiko Kiniwa on reasonable request.

## Abbreviations

LDH: Lactate dehydrogenase
CTC: Circulating tumor cells
ctDNA: Circulating tumor DNA
RECIST: Response Evaluation Criteria in Solid Tumors
CR: Complete response
PR: Partial response
SD: Stable disease
PD: Progressive disease
