# Supplementary material for: Usefulness of monitoring circulating tumor cells as a therapeutic biomarker in melanoma with BRAF mutation
Source: BMC Cancer. 2021 Mar 17;21:287. doi: 10.1186/s12885-021-08016-y (PMC7968258; doi:10.1186/s12885-021-08016-y)
Supplement: Supplementary file 1 — Additional file 1. [file 12885_2021_8016_MOESM1_ESM.docx]

**Supporting information**

**Methods**

**Cells and spike-in experiment**

Whole blood was collected in BD Vacutainer® Blood Collection Tubes (BD, Franklin Lakes, NJ, USA) from melanoma patients and healthy individuals. Immediately after, peripheral blood mononuclear cells (PBMCs) were isolated by gravity sedimentation and stored. To identify MART-1/gp100-positive and CD45-negative cells as melanoma cells, the intensities of fluorescently labelled markers and DAPI staining were determined in melanoma cell lines, which expressed both MART-1 and gp100, (888mel, 928mel, 501mel, 624mel, 397mel, MEL-2, and MEL-18), and PBMCs. A binary setting was determined to prioritize the removal of PBMCs. MART-1/gp100-positive and CD45-negative cells comprised 12.6–60.6% of the melanoma cells initially mixed with the peripheral blood in the binary setting (Fig. S1a). Furthermore, 888mel, 928mel, MEL-2, and MEL-18 were spiked serially (20/200/2000 or 50/500 cells) into PBMCs, and the cell suspension was added to the cell entrapment chamber, followed by counting of MART-1/gp100-positive and CD45-negative cells (Fig. S1b). The experiment was performed two to three times for each cell line.

**Cell capturing and DNA sequencing**

Captured cells from the cell entrapment chambers were singly aspirated from each well using glass capillaries with an inner diameter of 30 μm. The cell capturing process was described previously [1]. Isolated cells were collected into 10 μL water in tubes, followed by drying. Genomic DNA was extracted by incubating the cells for 16 hours at 50℃ in 3 μL lysis buffer (10 mM Tris-HCl (pH 8.3), 50 mM KCL, 4 mg/mL proteinase K and 3% Tween-20). Each DNA was directly amplified by nested PCR using inner and outer primers specific to *BRAF* exon15. The sequences of the outer primers were: (forward) 5’-CAT AAT GCT TGC TCT GAT AGG-3’ and (reverse) 5’-GGC CAA AAA TTT AAT CAG TGG A-3’. The cycling conditions were 22 cycles at 95℃ for 25 seconds, 55℃ for 25 seconds, and 72℃ for 30 seconds. The sequences of the inner primers were (forward) 5’-CAT AAT GCT TGC TCT GAT AGG-3’ and (reverse) 5’-TAG CCT CAA TTC TTA CCA TC-3’. The cycling conditions were 35 cycles at 95℃ for 20 seconds, 55℃ for 20 seconds, and 72℃ for 30 seconds. Nested PCR was conducted in 20 μL of reaction mixture using ExTaq (Takara, Shiga, Japan) in an iCycler (Bio-Rad Laboratories, Hercules CA, USA). Subsequently, PCR products were purified using the QIAquick PCR Purification Kit (Qiagen, Germantown, MD, USA). The sequence of the primer used for the forward reading reaction was 5’-TCA TAA TGC TTG CTC GAT AGG A-3’. Each sequencing reaction was performed using the BigDye Terminator v3.1 cycle sequence kit (Applied Biosystems, Foster City, CA, USA) and the products were loaded onto an ABI PRISM 3100 Genetic Analyzer (Applied Biosystems). Distilled water was used as a negative control for PCR and DNA sequencing.

**Figure legend**

**Figure S1.** Recovery of melanoma cells mixed with peripheral blood. (a) Cultured melanoma cells (888mel, 928mel, 501mel, 624mel, 397mel, MEL-2, and MEL-18) were mixed with the peripheral blood mononuclear cells. MART-1/gp100-positive and CD45-negative cells were detected as melanoma cells. (b) A representative spike-in experiment. 501mel (5, 50, and 500 cells) was mixed with peripheral blood mononuclear cells and the mixture was loaded into the cell entrapment chambers. The circles represent the number of detected cells. The dotted line represents the function y=x.

**Reference**

1. Morimoto A, Mogami T, Watanabe M, Iijima K, Akiyama Y, Katayama K, et al. High-Density Dielectrophoretic Microwell Array for Detection, Capture, and Single-Cell Analysis of Rare Tumor Cells in Peripheral Blood. PLoS One. 2015;10(6):e0130418.
