# Supplementary figures and images for: Usefulness of monitoring circulating tumor cells as a therapeutic biomarker in melanoma with BRAF mutation
Source: BMC Cancer. 2021 Mar 17;21:287. doi: 10.1186/s12885-021-08016-y (PMC7968258; doi:10.1186/s12885-021-08016-y)

## Slide 1
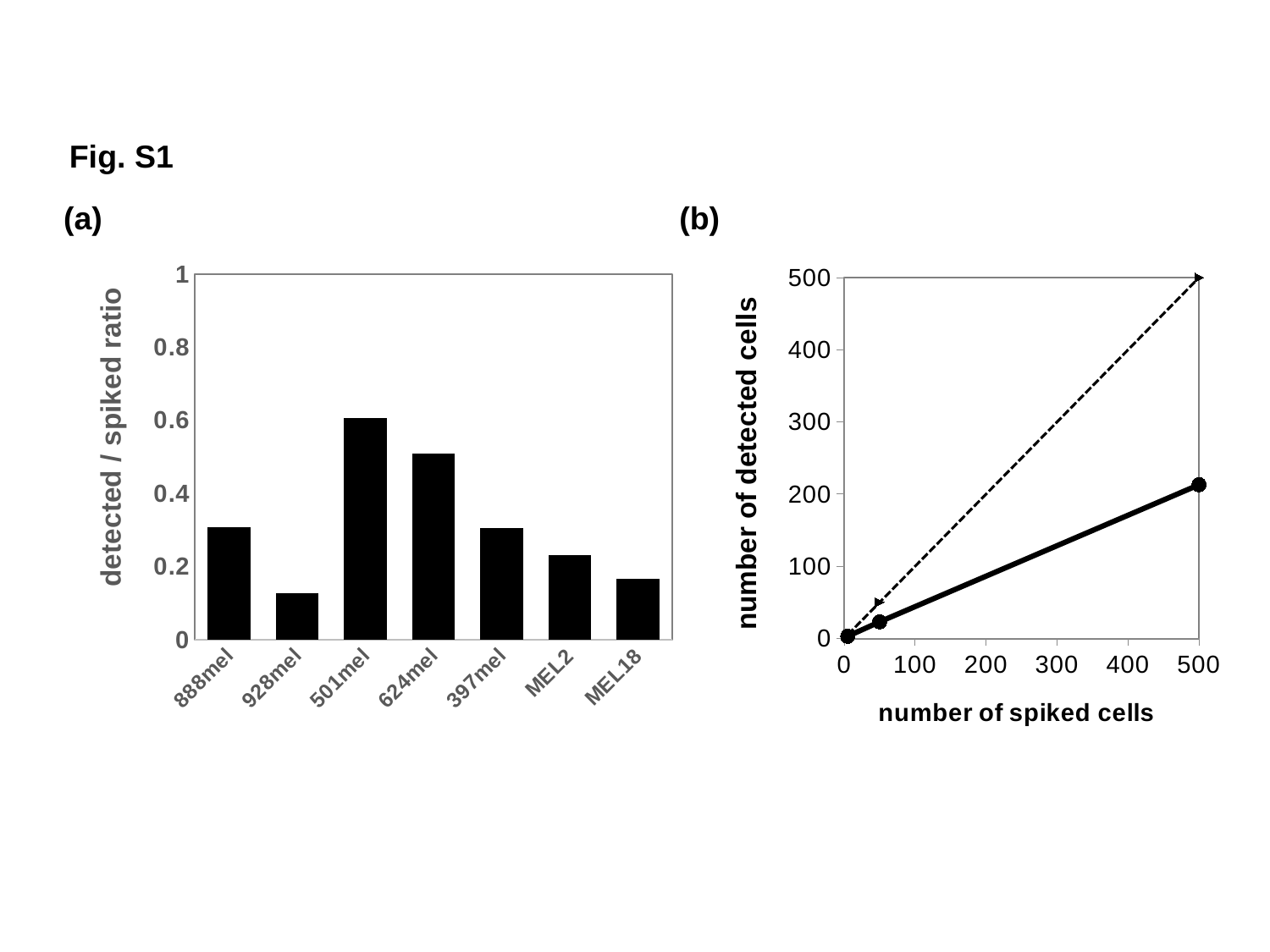

# Fig. S1
(a)
(b)
### Chart
| Category | % detected |
|---|---|
| 888mel | 0.3065 |
| 928mel | 0.126 |
| 501mel | 0.6055 |
| 624mel | 0.5095 |
| 397mel | 0.3045 |
| MEL2 | 0.2315 |
| MEL18 | 0.167 |
### Chart
| Category | | | |
|---|---|---|---|

Supplement: Supplementary file 3 — Additional file 3. [file 12885_2021_8016_MOESM3_ESM.pptx]
